# Rapid identification of *Lonicera japonica* via Proofman-LMTIA technology

**DOI:** 10.1038/s41598-025-91797-0

**Published:** 2025-03-06

**Authors:** Xiaodong Zhang, Caixia Li, Dejia Lan, Jinxin Liu, Deguo Wang

**Affiliations:** Food and Pharmacy College, Key Laboratory of Biomarker-based Rapid Detection Technology for Food Safety of Henan Province, College of Chemical and Materials Engineering, Xuchang University, Xuchang, 461000 China

**Keywords:** *Lonicera japonica*, Proofman-LMTIA, Rapid identification, Quality control, Biochemistry, Biological techniques, Molecular biology, Plant sciences

## Abstract

The study introduces a novel, rapid and precise method for identifying *Lonicera japonica* (JYH), an important medicinal herb, utilizing Proofman-LMTIA (Proofman probe-ladder melting temperature isothermal amplification) technology. Based on the 5.8 S-ITS2 rDNA sequence, specific primers and Proofman probes were designed to distinguish JYH from similar species, addressing the frequent market adulteration. The method’s optimal reaction conditions were established at 67 °C, ensuring high specificity. Sensitivity test demonstrated this method could detect as little as 10 pg/µL of JYH genomic DNA, with a detection limit of 1% (v/v) in adulteration experiments. The Proofman-LMTIA method successfully authenticated all eight market slice samples of JYH, six drugs and one solid beverage, achieving a 100% positive detection rate. This technique represents a significant advancement in medicinal material quality control, ensuring the JYH authenticity. These findings underscore the potential of the Proofman-LMTIA as a reliable, cost-effective, and user-friendly tool for real-time quality assessment in the herbal medicine industry.

**Supplementary Information:**

The online version contains supplementary material available at 10.1038/s41598-025-91797-0.

## Introduction

*Lonicera japonica* Thunb., commonly known as Jinyinhua (JYH), is a widely recognized medicinal herb in traditional Chinese medicine (TCM). As specified in the 2020 edition of the Chinese Pharmacopoeia, the dried flower buds or early blooming flowers of *L. japonica* are used for their renowned therapeutic properties, particularly in clearing heat, detoxifying, and dispersing wind-heat^1^. This herb is traditionally prescribed for conditions such as carbuncles, sore throat, erysipelas, dysentery, and warm diseases caused by wind-heat and fever^1^. In contrast, the Pharmacopoeia acknowledges several other species within the genus *Lonicera*, including *L. macranthoides*, *L. fulvotomentosa*, *L. confusa*, and *L. hypoglauca*, which are collectively referred to as Shanyinhua (SYH)^1^. These species share same therapeutic uses with JYH but differ significantly in chemical composition and medicinal efficacy^1^. JYH is particularly rich in chlorogenic acid and luteolin-7-O-glucoside, compounds that contribute to its superior efficacy in clearing heat and detoxifying^2^. In contrast, SYH species contain higher levels of chlorogenic acid but considerably lower amounts of luteolin-7-O-glucoside, along with other compounds like oleanolic acid glycosides that may cause adverse reactions when used in TCM injections^3–5^.

The higher medicinal value of JYH is reflected in its market price, which is approximately 2.5 times that of SYH during the same period^2,6^. This price disparity has led to widespread adulteration of JYH with the less expensive SYH in the processing of decoctions, pharmaceuticals, and food products^7,8^. The adulteration not only compromises the therapeutic quality of the products but also poses significant health risks to consumers.

Current methods for JYH identification, as outlined in the Chinese Pharmacopoeia, include morphological examination, microscopic identification, thin-layer chromatography (TLC), and high-performance liquid chromatography (HPLC)^1^. However, these techniques are often limited by subjectivity, the need for specialized equipment, and the requirement for skilled personnel​. Recent advances, such as Raman spectroscopy combined with deep learning and DNA barcoding based on the ITS2 sequence, have shown promise but remain hindered by the need for expensive equipment and extended detection times^9,10^.

Given the morphological similarities between JYH and SYH, particularly after processing, there is an urgent need for a rapid, cost-effective, and reliable method to authenticate JYH without the requirement for sophisticated equipment. Professor Wang Deguo’s team has addressed this need by developing the Proofman-LMTIA (Proofman probe-ladder melting temperature isothermal amplification) technology, which builds upon the loop-mediated isothermal amplification (LAMP) method​^11,12^. This technique offers a simpler, more accessible alternative to traditional methods, operating under constant temperature conditions without requiring complex instrumentation^13,14^.

This study applies Proofman-LMTIA technology for the rapid detection of JYH for the first time. Specific primers were designed on the basis of the differential regions of the 5.8 S-ITS2 sequences of JYH and SYH. The optimal temperature was first detected via a fluorescence-dye method, followed by the addition of Proofman probes, optimization of the reaction temperature, and determination of the specificity, sensitivity, and detection limits. Finally, the developed method was applied for the detection of commercial JYH decoction pieces, pharmaceuticals, and solid beverages, providing an effective solution for the authenticity identification of JYH medicinal materials, seedlings, food, pharmaceuticals, and health products.

## Materials and methods

### Plant materials

Standard samples of *Lonicera japonica* (JYH) and Shanyinhua (SYH, *L. macranthoides*) were provided by Yuzhou Housentang Traditional Chinese Medicine Co., Ltd. Standard sample of *L. hypoglauca* were provided by Wang Haichun, Chief of the Market Supervision and Administration Bureau of Pu’er City, Yunnan Province. Eight commercial slice samples of JYH were obtained from various markets. Six pharmaceutical products containing JYH were purchased from Henan Dazhongyuan Pharmaceutical Co., Ltd., China (Table 1). Additionally, Wanglaoji Honeysuckle Solid Beverage was purchased from Jingdong Online Shopping Mall (https://www.jd.com/). A plant genomic DNA extraction kit (DP305) was purchased from Tiangen Biochemical Technology (Beijing) Co., Ltd. The universal LMTIA reaction premix and 2×Mix were purchased from Dege Biotechnology (Shandong) Co., Ltd. GPV8 high-fidelity DNA polymerase (2 U/µL) was purchased from General Biology (Anhui) Co., Ltd.

**Table 1:** Experimental materials used in this study.

Sample	Scientific name	Source	Sample	Scientific name	Source
StandardLj	*L. japonica*	Yuzhou, China	Slice 7	*L. japonica*	Shandong, China
StandardLm	*L. macranthoides*	Hunan, China	Slice 8	*L. japonica*	Hebei, China
StandardLh	*L. hypoglauca*	Yunnan, China	Lianhua Qingwen Capsules	Contain *L. japonica*	Shijiazhuang Yiling Pharmaceutical Co., Ltd, China
Slice 1	*L. japonica*	Yuzhou, China	Pediatric Pharyngeal Flat Granules	Contain *L. japonica*	Lanzhou Heshengtang Pharmaceutical Co., Ltd, China
Slice 2	*L. japonica*	Yuzhou, China	Xiao’er Kechuanling Granules	Contain *L. japonica*	China Resources Sanjiu (Huangshi) Pharmaceutical Co., Ltd, China
Slice 3	*L. japonica*	Fengqiu, China	Compound Xiling Detoxification Capsules	Contain *L. japonica*	Shandong Hongjitang Pharmaceutical Group Co., Ltd, China
Slice 4	*L. japonica*	Fengqiu, China	Compound Honeysuckle Granules	Contain *L. japonica*	Heilongjiang Ussurijiang Pharmaceutical Co., Ltd. Harbin Branch, China
Slice 5	*L. japonica*	Bozhou, China	Yinqiaojiedu Honeyed Pill	Contain *L. japonica*	Henan Runhong Bencao Pharmaceutical Co., Ltd, China
Slice 6	*L. japonica*	Bozhou, China	Wanglaoji Honeysuckle Solid Beverage	Contain *L. japonica*	Guangdong Nanye Health Food High-end Manufacturing Co., Ltd, China

### Methods

#### Genomic DNA extraction

Slice samples were pulverized into a fine powder using a high-speed crusher and passed through a 60-mesh sieve. Genomic DNA was extracted using a plant genomic DNA extraction kit according to the manufacturer’s protocols. Drug and solid beverage samples were ground into power via mortar and pestle. The concentration and purity of the extracted DNA were determined using a NanoDrop One spectrophotometer (Thermo Fisher Scientific, Waltham, MA, USA). Only samples with A_260_/A_280_ ratios between 1.6 and 2.0 were used for subsequent LMTIA analysis. All extracted genomic DNA was stored at -20 °C until further use.

#### Cloning of 18 S-ITS1-5.8 S-ITS2 rDNA for JYH and SYH

A 200 µL PCR tube was prepared containing 25 µL of PrimeSTAR^®^ Max DNA Polymerase premix (2×, containing high-fidelity DNA polymerase, dNTP, and 2 × polymerase buffer), 2 µL of ITS-2F (10 µmol/L), 2 µL of ITS-3R (10 µmol/L), 4 µL of genomic DNA, and 17 µL of DEPC (diethyl pyrocarbonate)-treated water. The reaction conditions were: 98 °C for 10 s, 53 °C for 5 s, and 72℃ for 10 s, for a total of 30 cycles. Following PCR amplification, an A-tailing reaction was performed, and the amplified product was purified via agarose gel electrophoresis and gel recycle. The target fragment was ligated into a pMD19T vector and transformed into *Escherichia coli* DH5α competent cells using the heat-shock method. Positive colonies were screened on agar plates (1.5%, w/v) containing ampicillin (0.1 mg/mL), IPTG (0.024 mg/mL), and X-gal (0.04 mg/mL). Selected colonies were cultured, plasmids were extracted, and restriction enzyme digestion with *Eco* RI (Takara, Japan) confirmed the insertion of the target fragment. The plasmids were sent to Sangon Biotech (Shanghai) Co., Ltd. for sequencing, resulting in pMD19T-Lj18S-ITS1-5.8 S-ITS2 (pMD19T-LjDNA) for *L. japonica* and pMD19T-Lm18S-ITS1-5.8 S-ITS2 (pMD19T-LmDNA) for *L. macranthoides*.

#### Target sequence selection and LMTIA primer design

LMTIA primer design adhered to three key criteria^11^: (1) the melting temperature curve of the target sequence should display a ladder-like shape, (2) the GC content should range between 40% and 80%, and (3) the target sequence should be highly specific. The 5.8 S-ITS2 sequences of JYH and SYH were aligned using DNAMAN 7 software (version 7.0.2.716; Lynnon Biosoft, https://www.lynnon.com/dnaman.html) to identify regions of variation^15^. Primer3Plus (Primer3Plus Team, https://www.primer3plus.com) and Oligo 7 software (version 7.60; Molecular Biology Insights, Inc., https://www.oligo.net/) were used to design primers targeting the identified differential regions, including LjF1, LjB1, LjLF, and LjLB, with a Proofman probe^11^ (Table 2).

**Table 2:** Proofman-LMITA primers for *L. japonica*.

Primer name	Sequence (5′ to 3′)
LjF1	GACGCCCAGGCAGACGTTTTAATCCCGTGAACCATCGAGT
LjB1	CGTCACGCATCGCTTTTCCGCTCGCGACCCTG
LjLF	TAATGGCTTCGGGCGCAACT
LjLB	CCCCGCCCCGCCTCCCA
LjProbe	BHQ1-CCCCGCCCCGCT-JOE

#### LMTIA primer screening

SYBR Green I fluorescent dye method was used for initial primer screening. The following reagents were added to a 100 µL PCR tube: 5 µL of premix (containing *Bst* polymerase, dNTPs, and 2 × *Bst* polymerase buffer), 0.16 µL of LjF (10 µmol/L), 0.16 µL of LjB (10 µmol/L), 0.04 µL of LjLF (10 µmol/L), 0.04 µL of LjLB (10 µmol/L), 2 µL of pMD19T-LjDNA plasmid (10 ng/µL), and 2.60 µL of DEPC-treated water. The Gentier 96E fully automatic medical PCR analysis system was used with reaction temperatures set at 52 °C, 54 °C, 56 °C, and 58 °C over 40 cycles. Fluorescence signals were recorded every 90 s for a total of 40 times. The experimental group consisted of the JYH plasmid pMD19T-LjDNA (10 ng/µL), while the negative control was DEPC-treated water. The optimal primer set was selected based on the earliest amplification cycle.

#### Temperature optimization for Proofman-LMTIA

The Proofman-LMTIA reaction system consisted of 5 µL of 2× Mix, 0.4 µL of GPV8 high-fidelity DNA polymerase, 0.16 µL each of primers LjF1 and LjB1, 0.04 µL of primers LjLF and LjLB, 0.1 µL of 10 µmol/L Proofman probe, 2 µL of plasmid pMD19T-LjDNA template, and 2.10 µL DEPC-treated water. Reactions were performed at temperatures 59 °C, 61 °C, 63 °C, 64 °C, 65 °C, 66 °C, and 67 °C, and fluorescence signals were collected every 30 s for 10 cycles using the Gentier 96E system. The temperature with the fewest amplification cycles was selected as optimal.

#### Specificity experiment for Proofman-LMTIA

To evaluate the specificity of the Proofman-LMTIA method, 10 ng/µL of plasmid pMD19T-LjDNA was used as the positive control and 10 ng/µL of plasmid pMD19T-LmDNA and genomic DNA of *L. hypoglauca* were used as the negative controls. Reactions were performed with the optimized Proofman-LMTIA system, and fluorescence signals were recorded every 30 s for 60 cycles using the Gentier 96E system.

#### Sensitivity experiment for Proofman-LMTIA

The sensitivity of the Proofman-LMTIA method was assessed using serial dilutions of the pMD19T-LjDNA plasmid (10 ng/µL, 1 ng/µL, 100 pg/µL, 10 pg/µL, and 1 pg/µL). Reactions were carried out under optimal conditions, and each concentration was tested in triplicate. DEPC-treated water served as the negative control. Fluorescence signals were collected every 30 s for 40 cycles.

#### Detection limit experiment for Proofman-LMTIA

Simulated adulteration experiments were performed by preparing mixtures of pMD19T-LjDNA and pMD19T-LmDNA at JYH concentrations of 0%, 0.1%, 1.0%, and 5.0%. These mixtures were analyzed using the Proofman-LMTIA method at 67 °C, with fluorescence signals collected every 30 s for 40 cycles. Each experiment was performed in triplicate.

#### Market sample detection

Genomic DNA was extracted from eight market slice samples, six medicinal products containing honeysuckle, and one solid beverage. DNA quality was verified using a NanoDrop One spectrophotometer, and samples were tested using the established Proofman-LMTIA method under optimal conditions. Results were validated through comparison with positive and negative controls.

## Results

### 5.8S-ITS2 rDNA sequence analysis and primer design for JYH and SYH

**Fig. 1:**
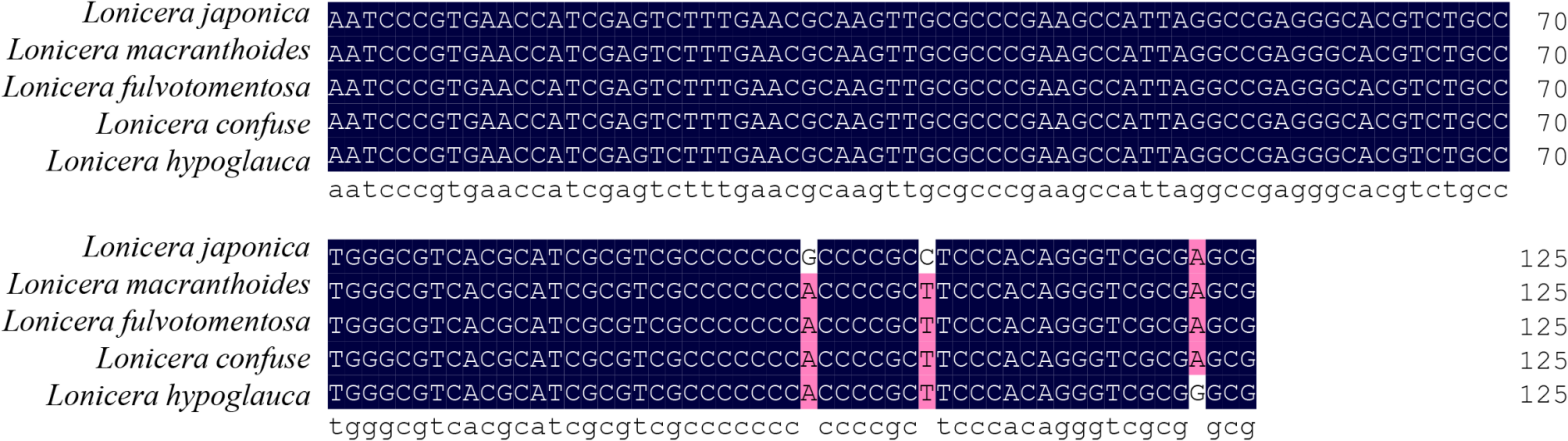
Alignment results of 5.8 S-ITS2 rDNA sequences for *Lonicera japonica*, *L. macranthoides*, *L. fulvotomentosa*, *L. confusa* and *L. hypoglauca*.

The 5.8 S-ITS2 rDNA sequences of JYH and SYH were aligned via DNAMAN7 software^15^. Significant sequence differences were identified between the five species, including three single nucleotide polymorphisms (SNPs) at positions 99G/A, 106 C/T, and 122 A/G (Fig. 1). These differential sites were used to design specific primers and Proofman probes following LMTIA primer design principles. The primers targeted regions with distinct ladder-shaped melting temperature curves, ensuring successfully amplification for JYH (Table 2, Figure S1).

### Cloning of 18 S-ITS1-5.8 S-ITS2 rDNA

**Fig. 2:**
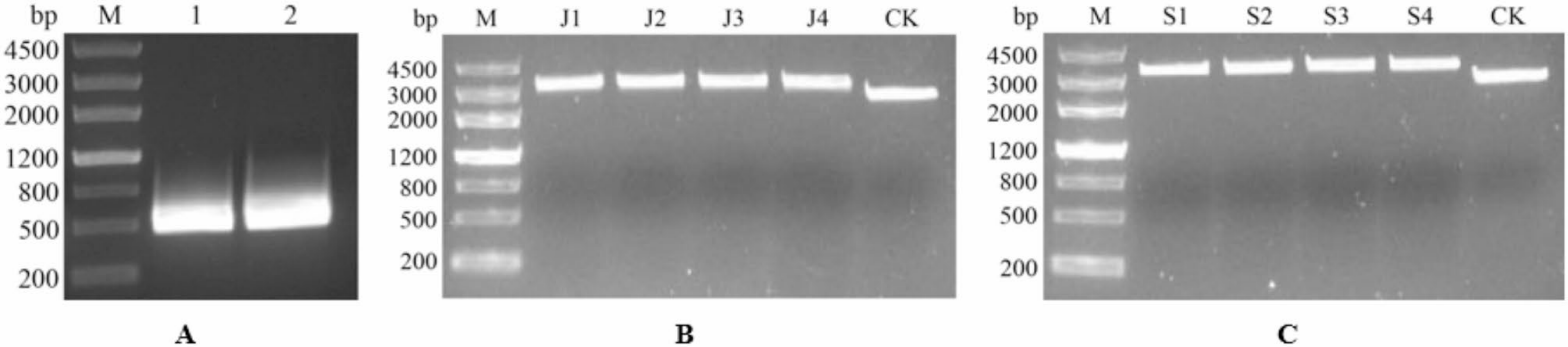
Cloning results of 18 S-ITS1-5.8 S-ITS2 rDNA for *L. japonica* and *L. macranthoides*. (**A**) PCR results of the target sequences; M: DNA standard; 1: *L. japonica*; 2: *L. macranthoides.* (**B**) Digestion of the pMD19T-Lj18 S-ITS1-5.8 S-ITS2 plasmid by *Eco* RI; M: DNA standard; J1-J4: plasmids from different clones; CK: pMD19T. (**C**) Digestion of the pMD19T-Lm18 S-ITS1-5.8 S-ITS2 plasmid by *Eco* RI; M: DNA standard; S1-S4: plasmids from different clones; CK: pMD19T.

PCR amplification of the 18 S-ITS1-5.8 S-ITS2 region from *L. japonica* and *L. macranthoides* produced fragments approximately 500 bp in length (Fig. 2A). Successful cloning of the target sequences into pMD19T vectors was confirmed through restriction enzyme digestion with *Eco* RI, which produced the expected banding patterns (Fig. 2B,C). Sequencing results verified the correct insertion of the 18 S-ITS1-5.8 S-ITS2 rDNA sequences into the plasmids, designated as pMD19T-LjDNA for JYH and pMD19T-LmDNA for SYH.

### Results of primer screening for the fluorescent dye LMTIA

**Fig. 3:**
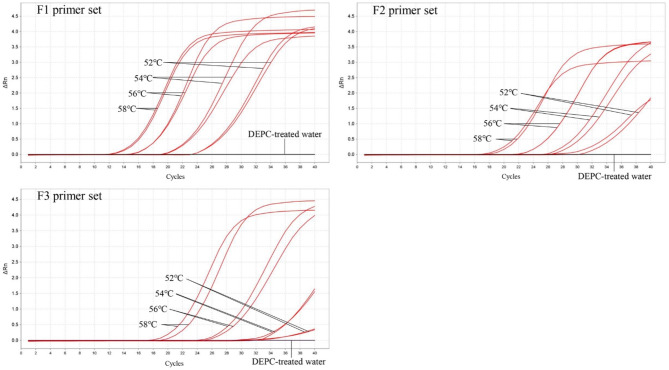
Primer screening results for *Lonicera japonica*.

Primer screening was performed using SYBR Green I fluorescence detection. Three sets of primers were tested at different temperatures (52 °C, 54 °C, 56 °C, and 58 °C), and the amplification cycles for the JYH plasmid pMD19T-LjDNA were compared. The F1 primer set demonstrated the earliest amplification at 58 °C, achieving significant amplification by the 12th cycle. In contrast, the F2 and F3 primer sets showed delayed amplification, starting from the 18th and 19th cycles, respectively (Fig. 3). Based on these results, the F1 primer set was selected for further testing due to its superior amplification efficiency at 58 °C.

### Results of temperature optimization for the Proofman-LMTIA

**Fig. 4:**
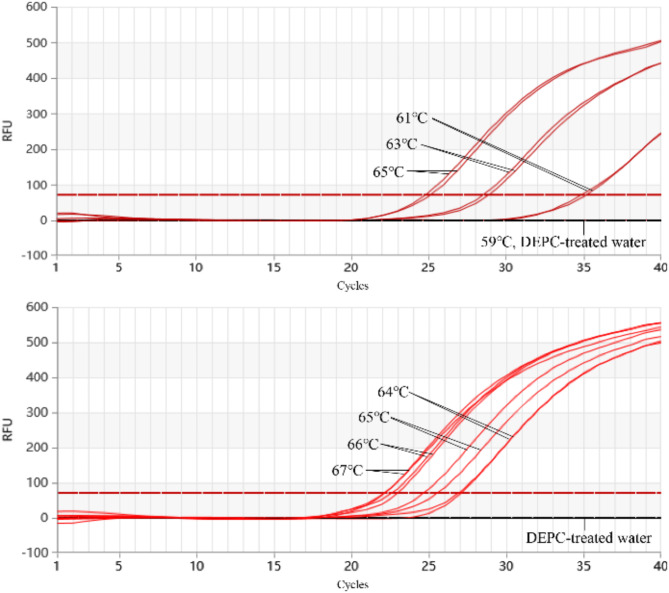
Results of the temperature optimization for the Proofman-LMTIA.

The optimal reaction temperature for Proofman-LMTIA was determined by testing a range of temperatures from 61 °C to 67 °C. The fastest amplification was observed at 67 °C, where the amplification cycle began at the 17th cycle. Lower temperatures (61 °C to 65 °C) resulted in progressively delayed amplification, with cycles starting between the 17.5th and 30th cycles (Fig. 4). The negative control and reactions conducted at 59 °C showed no amplification. Based on these findings, 67 °C was selected as the optimal temperature for subsequent experiments.

### Results of the specificity experiment for the Proofman-LMTIA

**Fig. 5:**
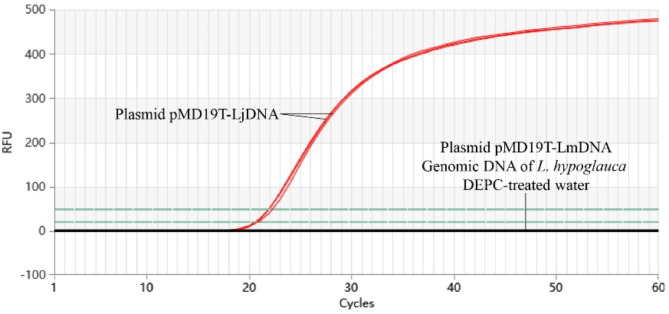
Specificity of Proofman-LMTIA for JYH. (1) Genomic DNA of JYH; (2) Genomic DNA of SYH.

To assess the specificity of the Proofman-LMTIA method, reactions were conducted using 10 ng/µL of the JYH plasmid pMD19T-LjDNA as the positive control and 10 ng/µL of the SYH plasmid pMD19T-LmDNA and genomic DNA of *L. hypoglauca* as the negative control. Amplification occurred exclusively in the presence of JYH plasmid DNA, confirming the high specificity of the method for JYH (Fig. 5).

### Results of the sensitivity experiment for Proofman-LMTIA

**Fig. 6:**
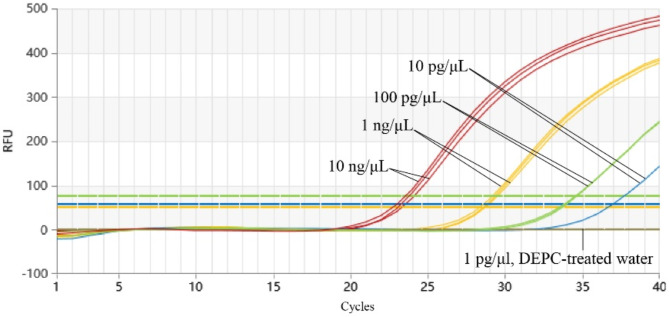
Sensitivity of Proofman-LMTIA for JYH detection.

The sensitivity of the Proofman-LMTIA method was evaluated by testing serial dilutions of the JYH plasmid pMD19T-LjDNA ranging from 10 ng/µL to 1 pg/µL. The method demonstrated reliable amplification at concentrations as low as 10 pg/µL. No amplification was observed at 1 pg/µL, establishing a sensitivity threshold of 10 pg/µL (Fig. 6).

### Results of the detection limit for Proofman-LMTIA

**Fig. 7:**
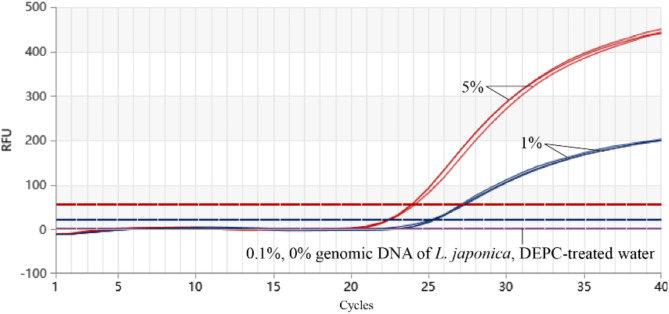
Detection limit of Proofman–LMTIA for JYH in adulteration experiment with SYH.

Mixtures of JYH and SYH plasmids were prepared with varying proportions of JYH plasmid pMD19T-LjDNA (0%, 0.1%, 1.0%, and 5.0%). Proofman-LMTIA successfully detected JYH at concentrations as low as 1% (v/v), demonstrating a detection limit of 1% (v/v) for the method (Fig. 7).

### Test results of market samples via Proofman-LMTIA

**Fig. 8:**
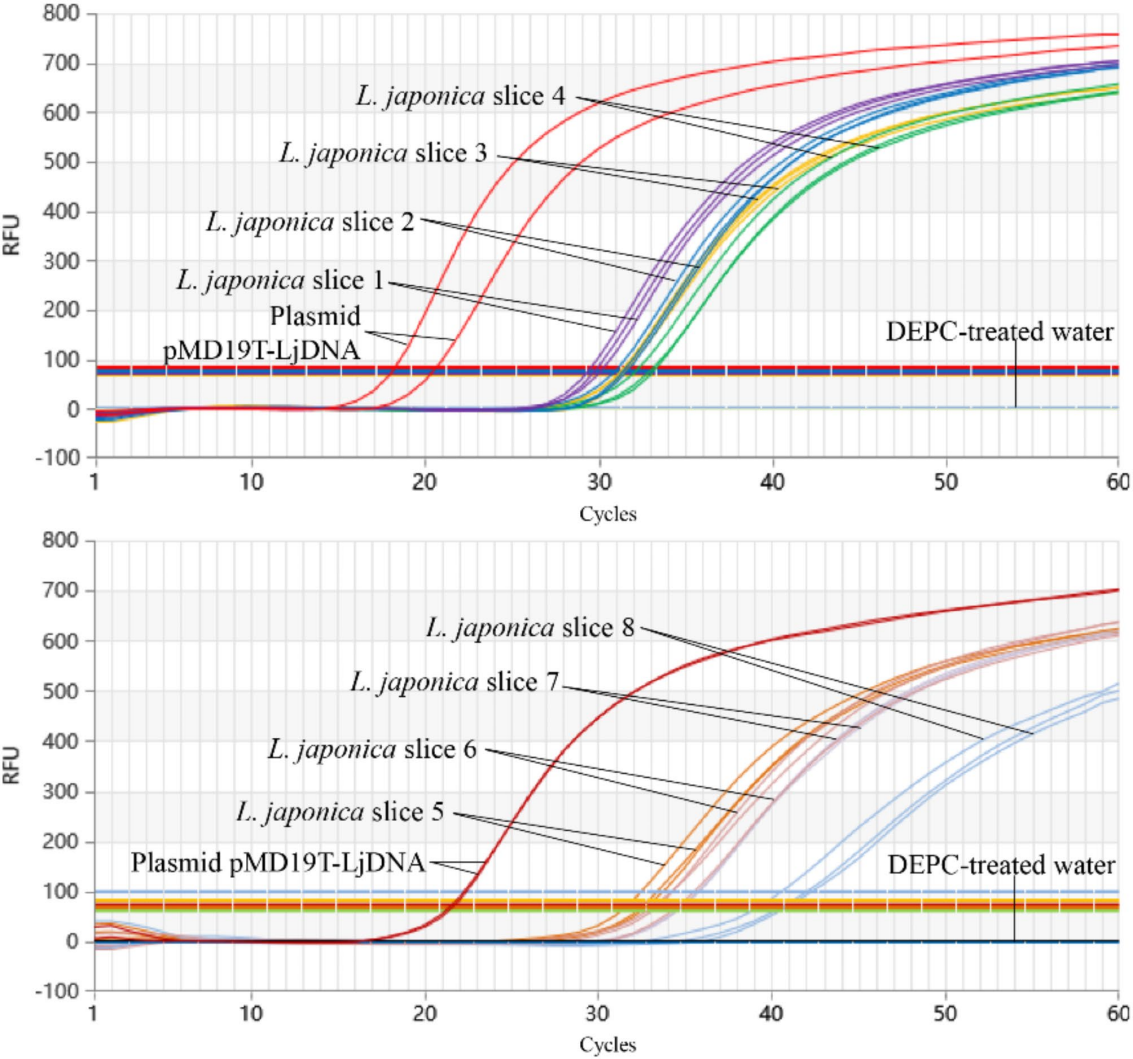
Detection of JYH slices in market using Proofman-LMTIA.

**Fig. 9:**
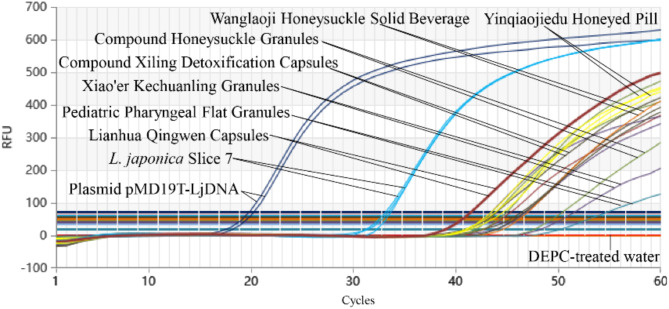
Detection of medicinal products containing honeysuckle components in market via Proofman-LMTIA.

Proofman-LMTIA was applied to genomic DNA extracted from eight commercial JYH slice samples, six JYH-containing medicinal products, and one solid beverage. All eight JYH slice samples were positively identified as JYH with a 100% detection rate (Fig. 8). Similarly, the six medicinal products and the solid beverage containing honeysuckle were confirmed to contain JYH, further validating the method’s reliability in real-world applications (Fig. 9).

## Discussion

The misidentification of JYH with SYH is a prevalent issue, particularly in processed TCMs such as powders, capsules, granules, and tablets. Traditional morphological and chemical identification methods often fall short in accurately distinguishing these species. Genomic DNA, as the material basis for species differences, offers a stable and reliable alternative, unaffected by material type or environmental factors. Utilizing specific genomic DNA sequences for species identification is an effective solution. However, existing DNA identification methods for JYH often require large sample sizes, lengthy detection times, and expensive equipment, and they necessitate skilled operators^10,16,17^.

In this study, we developed the Proofman-LMTIA method, which leverages LMTIA isothermal amplification technology and the specificity of Proofman probes. This method outperforms established techniques such as multiplex ligation-dependent probe amplification high-resolution melting (MLPA-HRM) and RNase H2-dependent PCR HRM (rhPCR-HRM)^18^ in terms of shorter detection time, higher specificity, and sensitivity. The Proofman-LMTIA method can complete genomic DNA extraction and authenticity determination within 1 h, making it highly suitable for identifying highly processed TCM products containing multiple herbal ingredients.

Specificity is a key aspect of nucleic acid detection. In our study, we identified the specific 5.8S-ITS2 sequences of JYH and SYH through literature search, database retrieval, and sequence alignment. These sequences were obtained via gene cloning and verified by first-generation sequencing. Unlike ordinary PCR, which relies solely on primer specificity, and MLPA-HRM and rhPCR-HRM^18^, which depend on probe specificity, the Proofman-LMTIA reaction employs specific primers to amplify a dumbbell structure. Subsequently, specific loop primers exponentially amplify this structure, providing the first level of specificity. Proofman probes bind to the specific products, and a deliberately designed mismatch base at the 3’ end is proofread and cut to release a luminous group, enabling fluorescence detection. This represents the second level of specificity at the single base level. Compared to typical isothermal amplification technologies like LAMP and recombinase polymerase amplification (RPA)^8,9^, Proofman-LMTIA significantly reduces false positives through dual specificity.

Sensitivity and detection limit are also critical in nucleic acid detection. The MLPA-HRM and rhPCR-HRM methods used by Mo et al. have a detection sensitivity of 100 ng/µL and detection limits of 10% and 5%, respectively^18^. In contrast, the Proofman-LMTIA method demonstrates a sensitivity of 10 pg/µL and a detection limit of 1% in JYH detection, which is 10 times higher than the aforementioned methods, highlighting its robustness and reliability in practical applications (Figs. 6 and 7). This result is comparable to the sensitivity of 10 pg/µL and detection limit of 1% in detecting sweet potato components in starch using Proofman-LMTIA^19^, but lower than the sensitivity of 1 pg/µL and detection limit of 0.1% in detecting *Panax quinquefolius* and *P. ginseng*^20^. This discrepancy may be due to the different DNA templates used in these experiments; in *P. quinquefolius* and *P. ginseng*, the template is genomic DNA with multiple copies of the 18 S rDNA target sequence, whereas in this study, the template is plasmid DNA containing 5.8 S-ITS2, or it may be caused by differences in DNA template purity.

The successful application of Proofman-LMTIA to market samples further underscores its practical utility. All tested JYH slice samples, pharmaceutical products, and beverages were accurately identified, achieving a 100% detection rate (Figs. 8 and 9). This demonstrates the method’s robustness and reliability in real-world settings, which is essential for ensuring the authenticity and quality of JYH products in the marketplace.

Despite these promising results, several limitations remain. Proofman-LMTIA, while highly specific and sensitive, is currently limited to qualitative detection, indicating the presence or absence of JYH rather than providing quantitative information on the amount of JYH in a given sample. This limitation could be addressed by developing a quantitative version of the method, for example, by introducing an internal reference gene, which would allow for the precise measurement of JYH concentrations in commercial products. Additionally, while the Proofman-LMTIA system is highly accessible, further integration with portable and automated platforms could increase its utility in field settings, enabling rapid on-site testing at production facilities, markets, and even in remote locations.

Future study should also explore the potential for multiplexing the Proofman-LMTIA system. By incorporating additional primers and probes of the fakes, it may be possible to simultaneously detect multiple target sequences, including not only JYH species but also other common adulterants such as SYH found in TCM products. Combining Proofman-LMTIA with machine learning algorithms could further enhance detection accuracy and streamline data analysis, making the method even more powerful for quality control in the herbal medicine industry.

## Conclusions

The Proofman-LMTIA method represents a significant step forward in the molecular identification of medicinal herbs, particularly JYH. It offers a simple, cost-effective, and reliable alternative to traditional and advanced identification techniques. By providing rapid, specific, and sensitive detection of JYH, Proofman-LMTIA addresses a critical need in the herbal medicine industry for effective quality control. Its successful application in this study emphasizes its potential for widespread adoption, improving the safety and authenticity of herbal products for consumers.

## Electronic supplementary material

Below is the link to the electronic supplementary material.

Supplementary Material 1

## Data Availability

The 5.8 S-ITS2 sequences utilized in this study include those from GenBank: *Lonicera japonica* (accession number: FJ372915.1), *L. macranthoides* (accession number: KF160915.1), *L. fulvotomentosa* (accession number: KF160913.1), *L. confusa* (accession number: KM258487.1) and *L. hypoglauca* (accession number: KM258490.1). The 18 S-ITS1-5.8 S-ITS2 sequence we cloned are *L. japonica* (accession number: PP218138.1), *L. macranthoides* (accession number: PQ475875).
