# Supplementary material for: Rapid identification of Lonicera japonica via Proofman-LMTIA technology
Source: Sci Rep. 2025 Mar 6;15:7913. doi: 10.1038/s41598-025-91797-0 (PMC11885523; doi:10.1038/s41598-025-91797-0)
Supplement: Supplementary file 1 — Supplementary Material 1 [file 41598_2025_91797_MOESM1_ESM.docx]

**Supplementary information**

**Supplementary Figures**


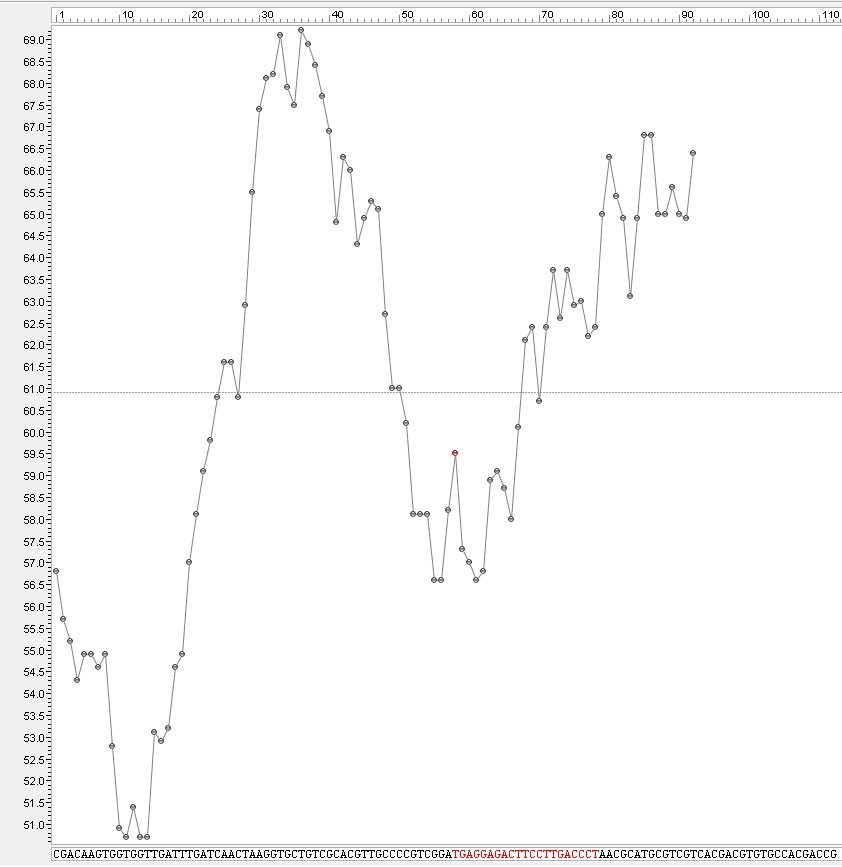


**Figure S1.** Ladder-shaped melting curve of the target sequence.

**Original Figures 2A, 2B and 2C**


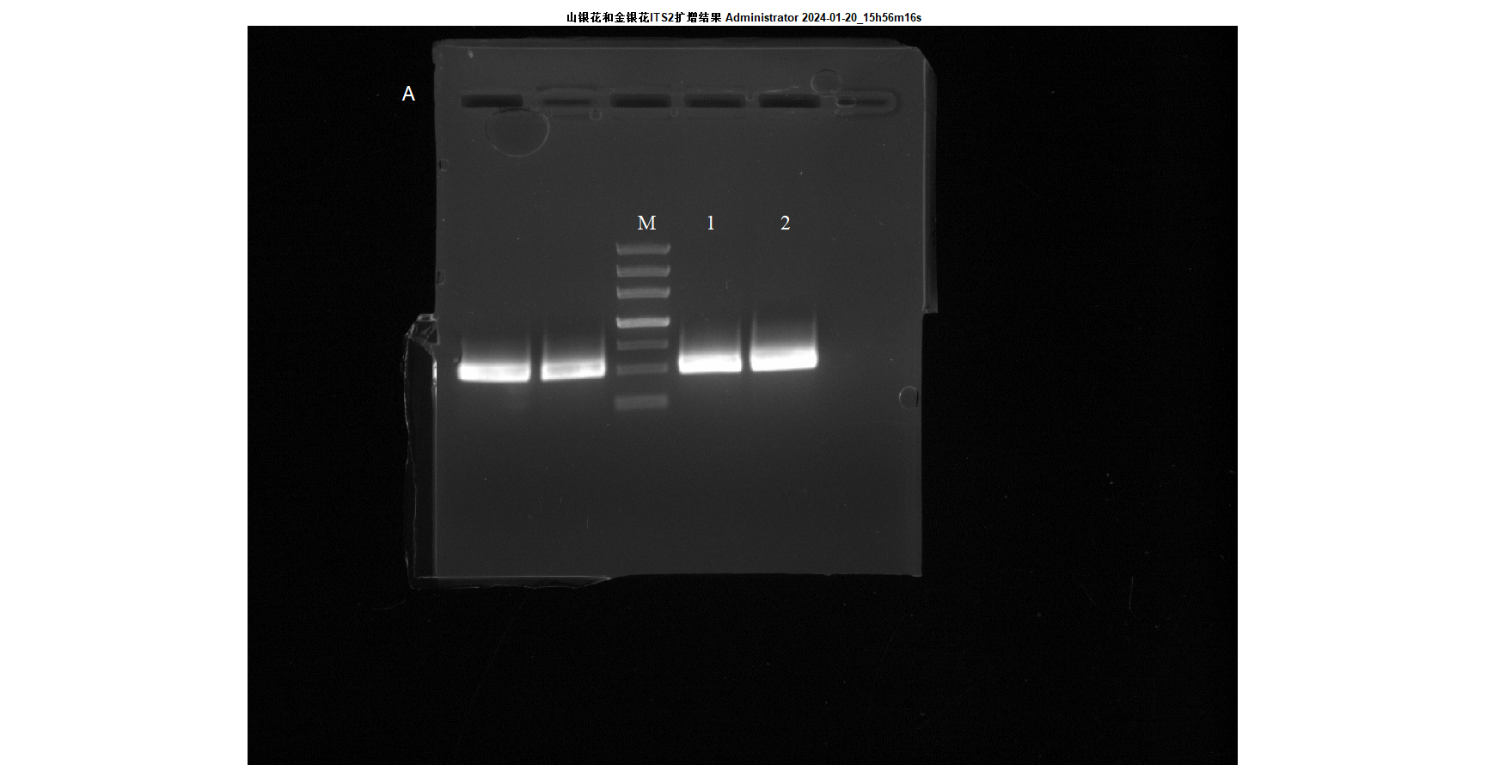


**Figure 2A.** PCR results of the target sequences. M: DNA standard; 1: *L. japonica*; 2: *L. macranthoides.*


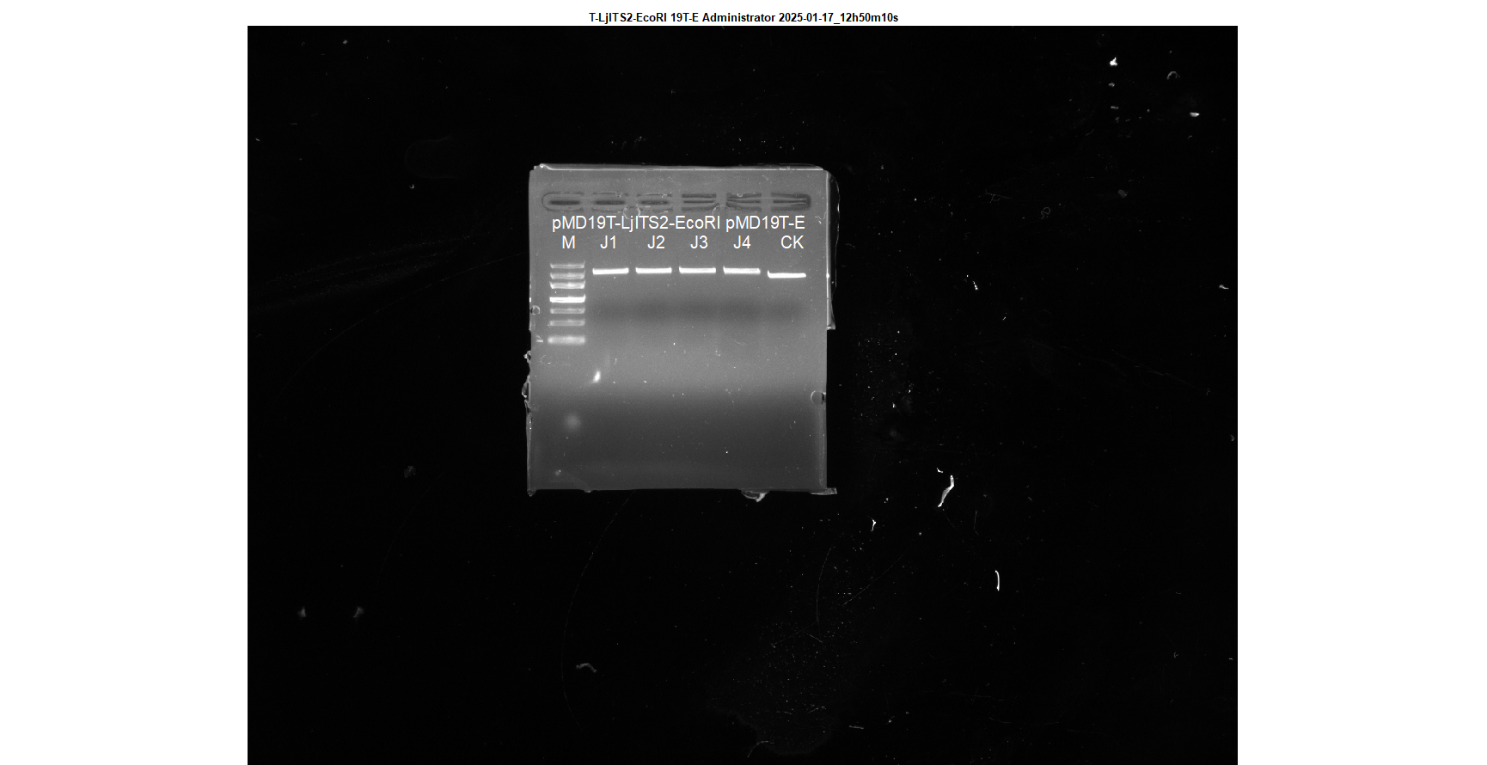


**Figure 2B.** Digestion of plasmid pMD19T-Lj18S-ITS1-5.8S-ITS2 by *Eco* RI. M: DNA standard; J1-J4: plasmids from different clones; CK: Digestion of plasmid pMD19T by *Eco* RI.


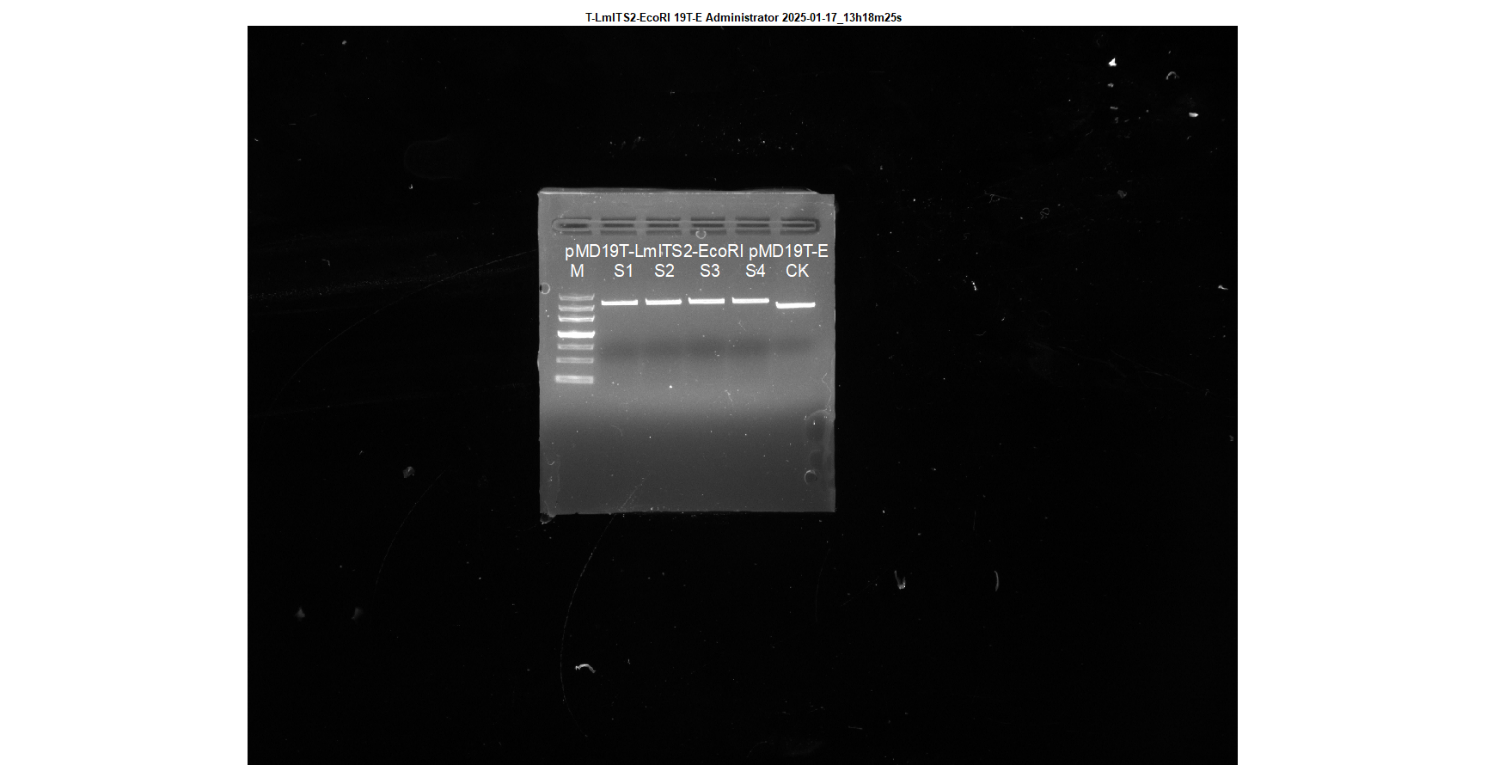


**Figure 2C.** Digestion of plasmid pMD19T-Lm18S-ITS1-5.8S-ITS2 plasmid by *Eco* RI. M: DNA standard; S1-S4: plasmids from different clones; CK: Digestion of plasmid pMD19T by *Eco* RI.

**Supplementary Files**

**Sequencing results for eight JYH slices**

>0001_32125012002601_(LJ1)_[ITS-2F]

GGGGGACTCGAGTCTTTGACGCAAGTTGCGCCCGAAGCCATTAGGCCGAGGGCACGTCTGCCTGGGCGTCACGCATCGCGTCGCCCCCCCGCCCCGCCTCCCACAGGGTCGCGAGCGGCGGGGGGCGCGGACAATGGCCCCCCGTGCCCCCGGGCGCGGCCGGCCCAAAATCGAGTCCCCGGCGGCGGACGTCACGACGAGTGGTGGTCGTAACATTCCTCTTATCGCGTCGTGCGGTTCCCCGTCGCCCGGGCGACCGAGTGACCCTGACGCGCCGTCGTACGACGGCGCTCCGACCGCGACCCCAGGTCAGGCGGGATTACCCGCTGAGTTTAAGCATATCAATAAGCGGAGGAAAAGAAACTTACAAGGATTCCCCTAGTAACGGCGAGCGAACCGGGAACAGCCCAGCTTGAGAATCGGGCGGCCTCGCCGTCCGAATTGTAGTCTGGCAAAGCGTCAA

>0002_32125012002602_(LJ2)_[ITS-2F]

GATGGGACTCGAGTCTTTGACGCAAGTTGCGCCCGAAGCCATTAGGCCGAGGGCACGTCTGCCTGGGCGTCACGCATCGCGTCGCCCCCCCGCCCCGCCTCCCACAGGGTCGCGAGCGGCGGGGGGCGCGGACAATGGCCTCCCGTGCCCCCGGGCGCGGCCGGCCCAAAATCGAGTCCCCGGCGGCGGACGTCACGACGAGTGGTGGTCGTAACATTCCTCTTATCGCGTCGTGCGGTTCCCCGTCGCCCGGGCGACCGAGTGACCCTGACGCGCCGTCGTACGACGGCGCTCCGACCGCGACCCCAGGTCAGGCGGGATTACCCGCTGAGTTTAAGCATATCAATAAGCGGAGGAAAAGAAACTTACAAGGATTCCCCTAGTAACGGCGAGCGAACCGGGAACAGCCCAGCTTGAGAATCGGGCGGCCTCGCCGTCCGAATTGTAGTCTGGA

>0003_32125012002603_(LJ3)_[ITS-2F]

GGATTACTCGAGTCTTTGACGCAAGTTGCGCCCGAAGCCATTAGGCCGAGGGCACGTCTGCCTGGGCGTCACGCATCGCGTCGCCCCCCCGCCCCGCCTCCCACAGGGTCGCGAGCGGCGGGGGGCGCGGACAATGGCCCCCCGTGCCCCCGGGCGCGGCCGGCCCAAAATCGAGTCCCCGGCGGCGGACGTCACGACGAGTGGTGGTCGTAACATTCCTCTTATCGCGTCGTGCGGTTCCCCGTCGCCCGGGCGACCGAGTGACCCTGACGCGCCGTCGTACGACGGCGCTCCGACCGCGACCCCAGGTCAGGCGGGATTACCCGCTGAGTTTAAGCATATCAATAAGCGGAGGAAAAGAAACTTACAAGGATTCCCCTAGTAACGGCGAGCGAACCGGGAACAGCCCAGCTTGAGAATCGGGCGGCCTCGCCGTCCGAATTGTAGTCTGCAAAAGCGTCA

>0004_32124012501777_(B1)_[M13+]

ATGCGATACTTGGTGTGAATTGCAGAATCCCGTGAACCATCGAGTCTTTGAACGCAAGTTGCGCCCGAAGCCATTAGGCCGAGGGCACGTCTGCCTGGGCGTCACGCATCGCGTCGCCCCCCCGCCCCGCCTCCCACAGGGTCGCGAGCGGCGGGGGGCGCGGACAATGGCCCCCCGTGCCCCCGGGCGCGGCCGGCCCAAAATCGAGTCCCCGGCGGCGGACGTCACGACGAGTGGTGGTCGTAACATTCCTCTTATCGCGTCGTGCGGTTCCCCGTCGCCCGGGCGACCGAGTGACCCTGACGCGCCGTCGTACGACGGCGCTCCGACCGCGACCCCAGGTCAGGCGGGATTACCCGCTGAGTTTAAGCATATCAATAAGCGGAGGAAAAGAAACTTACAAGGATTCCCCTAGTAACGGCGAGCGAACCGGGAACAGCCCAGCTTGAGAATCGGGCGGCCTCGCCGTCCGAATTGTAGTCTGGAGAAGCGTC

>0008_32124012501781_(C2)_[M13+]

ATGCGATACTTGGTGTGAATTGCAGAATCCCGTGAACCATCGAGTCTTTGAACGCAAGTTGCGCCCGAAGCCATTAGGCCGAGGGCACGTCTGCCTGGGCGTCACGCATCGCGTCGCCCCCCCGCCCCGCCTCCCACAGGGTCGCGAGCGGCGGGGGGCGCGGACAATGGCCCCCCGTGCCCCCGGGCGCGGCCGGCCCAAAATCGAGTCCCCGGCGGCGGACGTCACGACGAGTGGTGGTCGTAACATTCCTCTTATCGCGTCGTGCGGTTCCCCGTCGCCCGGGCGACCGAGTGACCCTGACGCGCCGTCGTACGACGGCGCTCCGACCGCGACCCCAGGTCAGGCGGGATTACCCGCTGAGTTTAAGCATATCAATAAGCGGAGGAAAAGAAACTTACAAGGATTCCCCTAGTAACGGCGAGCGAACCGGGAACAGCCCAGCTTGAGAATCGGGCGGCCTCGCCGTCCGAATTGTAGTCTGGAGAAGCGTC

>0010_32124012501783_(D1)_[M13+]

ATGCGATACTTGGTGTGAATTGCAGAATCCCGTGAACCATCGAGTCTTTGAACGCAAGTTGCGCCCGAAGCCATTAGGCCGAGGGCACGTCTGCCTGGGCGTCACGCATCGCGTCGCCCCCCCGCCCCGCCTCCCACAGGGTCGCGAGCGGCGGGGGGCGCGGACAATGGCCCCCCGTGCCCCCGGGCGCGGCCGGCCCAAAATCGAGTCCCCGGCGGCGGACGTCACGACGAGTGGTGGTCGTAACATTCCTCTTATCGCGTCGTGCGGTTCCCCGTCGCCCGGGCGACCGAGTGACCCTGACGCGCCGTCGTACGACGGCGCTCCGACCGCGACCCCAGGTCAGGCGGGATTACCCGCTGAGTTTAAGCATATCAATAAGCGGAGGAAAAGAAACTTACAAGGATTCCCCTAGTAACGGCGAGCGAACCGGGAACAGCCCAGCTTGAGAATCGGGCGGCCTCGCCGTCCGAATTGTAGTCTGGAGAAGCGTC

>0014_32124012501787_(E4)_[M13+]

ATGCGATACTTGGTGTGAATTGCAGAATCCCGTGAACCATCGAGTCTTTGAACGCAAGTTGCGCCCGAAGCCATTAGGCCGAGGGCACGTCTGCCTGGGCGTCACGCATCGCGTCGCCCCCCCGCCCCGCCTCCCACAGGGTCGCGAGCGGCGGGGGGCGCGGACAATGGCCCCCCGTGCCCCCGGGCGCGGCCGGCCCAAAATCGAGTCCCCGGCGGCGGACGTCACGACGAGTGGTGGTCGTAACATTCCTCTTATCGCGTCGTGCGGTTCCCCGTCGCCCGGGCGACCGAGTGACCCTGACGCGCCGTCGTACGACGGCGCTCCGACCGCGACCCCAGGTCAGGCGGGATTACCCGCTGAGTTTAAGCATATCAATAAGCGGAGGAAAAGAAACTTACAAGGATTCCCCTAGTAACGGCGAGCGAACCGGGAACAGCCCAGCTTGAGAATCGGGCGGCCTCGCCGTCCGAATTGTAGTCTGGAGAAGCGTC

>0018_32124012501791_(F7)_[M13+]

ATGCGATACTTGGTGTGAATTGCAGAATCCCGTGAACCATCGAGTCTTTGAACGCAAGTTGCGCCCGAAGCCATTAGGCCGAGGGCACGTCTGCCTGGGCGTCACGCATCGCGTCGCCCCCCCGCCCCGCCTCCCACAGGGTCGCGAGCGGCGGGGGGCGCGGACAATGGCCCCCCGTGCCCCCGGGCGCGGCCGGCCCAAAATCGAGTCCCCGGCGGCGGACGTCACGACGAGTGGTGGTCGTAACATTCCTCTTATCGCGTCGTGCGGTTCCCCGTCGCCCGGGCGACCGAGTGACCCTGACGCGCCGTCGTACGACGGCGCTCCGACCGCGACCCCAGGTCAGGCGGGATTACCCGCTGAGTTTAAGCATATCAATAAGCGGAGGAAAAGAAACTTACAAGGATTCCCCTAGTAACGGCGAGCGAACCGGGAACAGCCCAGCTTGAGAATCGGGCGGCCTCGCCGTCCGAATTGTAGTCTGGAGAAGCGTC
